# Editorial: Advances in Combination Tumor Immunotherapy

**DOI:** 10.3389/fonc.2015.00198

**Published:** 2015-09-22

**Authors:** Michael A. Curran, Bernard A. Fox, William L. Redmond

**Affiliations:** ^1^Department of Immunology, UT MD Anderson Cancer Center, Houston, TX, USA; ^2^Providence Portland Medical Center, Robert W. Franz Cancer Research Center, Earle A. Chiles Research Institute, Portland, OR, USA

**Keywords:** immunotherapy, combination therapy, OX40, 4-1BB, checkpoint blockade

In the process of evolving from a normal cell into a malignant cancer, tumors accumulate numerous genetic aberrations, which should render them highly visible and susceptible to immune destruction, yet the unaided immune system fails to eliminate cancer. Decades of research has finally culminated in an ever-increasing understanding of the mechanisms by which tumors evade and suppress host immunity. With this understanding has come a renaissance in cancer therapy as immunotherapeutic interventions, which augment tumor-specific responses and block suppressive pathways on which tumors depend to maintain their immune privilege, have shown increasing efficacy in the clinic. The unprecedented therapeutic success of T cell checkpoint blockade and T cell adoptive cell therapy approaches in solid tumors, such as melanoma, and in certain leukemias led to Cancer Immunotherapy being named “Breakthrough of the Year” by Science magazine in 2013 (1).

Antibodies, which block the T cell co-inhibitory receptors, CTLA-4 and PD-1, have now achieved FDA approval for melanoma and lung cancer (PD-1), and numerous other immunotherapeutics are demonstrating efficacy in clinical trials. These drugs have introduced the hope of curative outcomes for metastatic cancer patients for whom benefit was previously measured in weeks to months of extended survival (2). The reality, however, is that <10% of melanoma patients achieve complete responses to these therapies, fewer than 5% of lung cancer patients do, and for many solid tumors, such as pancreatic or colorectal cancer, complete responses are almost never observed. This shift in the goal of immunotherapy from demonstration of therapeutic efficacy toward achieving complete responses in a majority of patients has highlighted the necessity for combining immunotherapeutics with similar interventions, as well as with more traditional oncology therapies, in order to achieve synergistic outcomes.

This special issue on “Advances in Combination Tumor Immunotherapy” highlights both emerging clinical immunotherapy combinations, promising future combinations centered on T cell co-stimulatory agonist antibodies based on extensive pre-clinical data, the potential of augmenting immunotherapy through combination with non-immune-based therapies, and the combinations of immune and non-immune therapies moving forward for tumors of specific histologies (e.g. prostate, AML). Combination blockade of CTLA-4 and PD-1 in malignant melanoma has yielded objective response rates over 55% with nearly 90% of patients surviving at 2 years versus a 2-year survival in this population of <20% as recently as 2010. Callahan and colleagues review the efficacy and toxicity of this potent combination of checkpoint blockade antibodies in trials of melanoma, renal cell carcinoma, and non-small cell lung cancer in their article “CTLA-4 and PD-1 pathway blockade: combinations in the clinic” (3). As this is likely to become the first FDA approved combination of immunomodulatory antibodies, understanding its clinical application as well as its potential and limitations when applied across multiple tumor histologies will provide a template for both assembling and judging the efficacy of future immune combinations.

Antibodies, which activate tumor-specific T cell responses by blocking co-inhibitory checkpoint receptors, have rapidly advanced to FDA approval, while those that act by activating co-stimulatory receptors to increase T cell effector function, proliferation, and survival have moved more slowly through clinical trials. Promising data from ongoing Phase I trials, coupled with potent enhancement of checkpoint antibodies in pre-clinical studies, predicts a critical role for these agonist antibodies in future combination therapies. Antibodies, which activate the tumor-necrosis factor receptor superfamily member (TNFRSF) OX40 (CD134), drive potent anti-tumor immune responses both alone and in combination with checkpoint blockade in pre-clinical models. In their article “OX40 Agonists and Combination Immunotherapy: Putting the Pedal to the Metal,” Linch et al. highlight the extensive pre-clinical data on this promising new class of agents as well as the rationale for combining OX40 antibodies with numerous other therapies (4). Importantly, they emphasize the very mild safety profile of OX40 agonists, which make them appealing both in combination studies and in treating patients with earlier stages of disease. 4-1BB (CD137) is another TNFRSF member related to OX40 for which agonist antibodies have been in clinical trials for nearly a decade. Bartkowiak and colleagues highlight the capacity of 4-1BB agonists, alone or in a variety of combination strategies, to cure tumors of virtually every lineage in murine models in their article “4-1BB Agonists: Multi-Potent Potentiators of Tumor Immunity” (5). The immense clinical potential of 4-1BB activation has yet to be realized due to the propensity of these antibodies to cause dose-limiting liver inflammation; however, recent discoveries may point to a path forward for these drugs in specific combinations, which enhance their efficacy while minimizing their side effects.

Traditional therapies, which increase the access of effector lymphocytes to the tumor parenchyma and augment the presentation of tumor-associated antigens, will be invaluable as part of combinations, which promote complete therapeutic responses. Vatner et al. describe how “Combinations of Immunotherapy and Radiation in Cancer Therapy” work together to diminish immune suppressive stromal populations in the microenvironment, enhance tumor antigen presentation, and increase access of T cells to irradiated lesions where they can be primed to seek out other, non-irradiated lesions, in order to mediate abscopal responses (6). Similarly, Heninger and colleagues describe how “Augmenting Antitumor Immune Responses with Epigenetic Modifying Agents” can form the backbone of a promising new approach to combination immunotherapy (7). Targeted modulation of epigenetic mechanisms of gene regulation can render tumors more immunogenic, stromal populations less suppressive, and increase the potency of immune effectors, yet the targets of these compounds are broad and care must be taken to select agents that have a net beneficial effect on tumor immune elimination.

The first FDA approved tumor vaccine targets prostate cancer, yet its effects are modest and immunotherapeutic combinations are sorely needed for castration-resistant disease. Burotto et al. propose many innovative combinations of immune and traditional therapies for prostate cancer as well as reviewing the pre-clinical and clinical experience with these combinations to date in their article “Exploiting Synergy: Immune-Based Combinations in the Treatment of Prostate Cancer” (8). Clinical development of immunotherapies targeting Acute Myeloid Leukemia has lagged despite promising pre-clinical studies highlighting a diverse array of vaccine, checkpoint, and innate combinations. Curran et al. describe the progress to date as well as identifying the combinations with the greatest clinical potential in their article “Targeting the Innate Immune System as Immunotherapy for Acute Myeloid Leukemia” (9).

Only through therapeutic combinations will we reach the goal of delivering curative cancer responses to a majority of patients. The articles in this issue describe several promising pathways along the road to achieving this goal.

## Conflict of Interest Statement

The authors declare that the research was conducted in the absence of any commercial or financial relationships that could be construed as a potential conflict of interest.
